# Parental drinking, mental health and educational level, and offspring’s subsequent prescription drugs treatment for sleep problems. A longitudinal HUNT survey and registry study

**DOI:** 10.1186/s12889-023-16301-7

**Published:** 2023-07-18

**Authors:** Ingunn Olea Lund, Njål Andersen, Helga Ask, Jasmina Burdzovic Andreas

**Affiliations:** 1 Norwegian Institute of Public Health, PO Box 222, Skøyen, Oslo, 0213 Norway; 2 Department of Psychology, Faculty of Social Sciences, University of Oslo, PO Box 1094, Blindern, Oslo, 0317 Norway; 3 Department of International Business, NTNU Ålesund, Postboks, Ålesund, 1517, 6025 Norway

**Keywords:** Parent, Alcohol, Mental health, Socioeconomic status, Adolescent, Sleep, Medication, Prescription drug

## Abstract

**Background:**

Parental drinking, mental health and family socioeconomic status are all associated with offspring sleep problems, but there is a paucity of research that considers the effect of risk factors, as they co-occur within and across families. Also, sleep problems are closely linked with mental health problems. Disentangling the effects on one or the other are important. We examined whether parental risk constellations are differently associated with offspring’s subsequent prescription drug use for sleep problems during nine years with or without prescription drug use for anxiety and/or depression.

**Methods:**

The sample included 8773 adolescent offspring of 6696 two-parent families who participated in the Nord-Trøndelag Health Study in Norway. The exposures were five parental risk constellations, previously identified via Latent Profile Analysis, characterized by drinking frequencies and quantities, mental health, and years of education. The outcomes were dispensed prescription drugs in offspring during 2008–2016 for (a) only sleep problems (b) sleep problems and anxiety/depression or (c) only anxiety/depression. We used multinomial logistic regression to model the odds of the outcomes.

**Results:**

Compared to the overall low-risk parental constellation, none of the risky constellations were significantly associated with increased risk of being dispensed prescription drugs only for sleep problems. Offspring from two different risk profiles were at increased risk for being dispensed both sleep and anxiety/depression prescription drugs. These were parental profiles marked by (1) low education, symptoms of mental health problems and weekly binge drinking in both parents (OR 1.90, CI = 1.06;3.42); and (2) frequent heavy drinking in both parents and symptoms of mental health problems in fathers (OR 3.32, CI = 1.49;7.39). Offspring from the risk profile with lowest parental education had increased risk of only anxiety/depression prescription drugs (OR 1.25, CI = 1.05;1.49).

**Conclusion:**

Our findings suggest that parental risk constellations are not associated with increased risk of offspring receiving sleep medications without also receiving anxiety/depression medications, as two risk constellations were associated with increased risk of dispensation of both sleep and anxiety/depression prescription drugs. Receiving both may be an indication of severity. The findings underscore the importance of including measures of mental health problems when investigating sleep problems to avoid misattribution of effects.

## Introduction

Although the need for sleep is universal, getting enough, and high-quality, sleep is particularly important during adolescence, a period with rapid physical, cognitive, and emotional development [1, 2]. Between 18 and 30% of adolescents and young adults in Norway experience insomnia [3], and 3.3% suffer from delayed sleep-wake phase disorder, that is, they struggle to fall asleep at the normal time and find it difficult to wake up [4]. Too little or poor sleep may negatively impact physical and mental health [5–7], contribute to alcohol and drug related problems [8], and result in poorer attendance and lower grades at school [9–11], sickness absence [12], as well as traffic and work accidents [13, 14]. Although the Norwegian national guidelines for treatment of sleep problems recommend cognitive behavior therapy as first-line treatment over prescription drugs [15], prescription drugs are by far the most frequently used treatment for sleep problems in Norway [15, 16], and therefore the focus of the current study. Prior research has shown that some parental risk constellations are associated with subsequent treatment for anxiety/depression in offspring [17]. Here we build on and extend that research by addressing whether some parental risk constellations are associated with subsequent prescription drugs treatment for sleep problems in offspring. We disentangle the role of parental risk constellations in predicting treatment for sleep problems as a separate entity from anxiety/depression, and treatment of sleep problems that precede, co-occur, or follow treatment for anxiety/depression.

A study based on cross-sectional data, assessed children’s sleep with actigraphs over 7 nights, showed that parental problem drinking was associated with shorter sleep duration, reduced sleep efficiency, and greater long wake episodes in offspring [18]. Further, a longitudinal study showed an association between parental problem drinking and reduced sleep duration and sleep efficiency in offspring over time [19]. Poor parental mental health and low socioeconomic status (SES) are also both associated with offspring sleep problems [20–26], and these three risk factors - mental health, low SES and problem drinking - often co-occur [27, 28]. To date, most studies have focused on single parental risk factors, a tendency that may lead to underreporting of true risk. A combination of risk factors, including risk factors below clinical level, may accumulate, and represent significant risk. Studies that examine single risk factors, may present elevated risk, or obscure actual risk [29]. Rather than studying risk factors in isolation, it is more informative and nuanced to consider risk factors together, as they co-occur within and across families.

Historically, sleep problems were mainly considered as symptoms of depression or anxiety [30, 31]. Indeed, problems falling asleep, staying asleep, and restless sleep are common symptoms of anxiety and depression and listed along with the other symptoms of these disorders in diagnostic manuals [32]. However, sleep problems are also considered as separate disorders [30]. The sleep related complaints in the research diagnostic criteria for insomnia (i.e. difficulty initiating sleep, difficulty maintaining sleep, waking up too early or sleep that is chronically nonrestorative or of poor quality) largely overlap with the sleep related symptoms of anxiety and depression [33]. In order to separate sleep problems that occurs independently from sleep problems that are symptoms of anxiety and depression, some sleep studies control for anxiety/depression [34, 35]. However, many studies fail to do so [10, 19, 36, 37]. As the etiology for sleep problems may differ from the etiology of anxiety/depression, it is necessary to account for the latter when examining sleep problems in general, namely those not associated with anxiety/depression. Failure to do so may lead to possible conflated results or masked true effects. Further, given the bidirectional relationship between sleep and anxiety/depression, extensive follow-up time is necessary to identify cases where sleep problems occur without instances of anxiety/depression. Among Norwegian children and adolescents, the prevalence of diagnosed depression is between 0.1 and 2.7% [38–42]. For anxiety disorders, the prevalence is between 1.5 and 5.3% [38, 39, 42, 43].

To date, most studies are cross-sectional or have short follow-up period, see e.g. [18–20, 26], adding uncertainty as to the problems that are captured during data collection; sleep problems, anxiety/depression, or both. Further, most previous research has focused on self-reported symptoms of sleep problems and anxiety/depression, or sleep data from actigraphs, which consequently has formed the basis of the current knowledge on the association between parental risk constellations and offspring sleep problems. Some studies also specifically exclude cases where offspring present with a diagnosed sleep disorder [18, 19] The current study contributes to and extend knowledge through use of a different approach, namely pharmacological treatment for sleep problems with and without preceding, co-occurring, or following prescription drug use for anxiety/depression.

The prevalence of sleep problems in young people, and the adverse outcomes associated with it, underscore the need for reliable knowledge about its predictors. We examine the effect of parental risk factors on subsequent prescription drug use for sleep problems in offspring as adolescents and young adults. We use a large cohort study, where both parents and offspring provide information on key variables through survey self-report at baseline. We follow offspring prospectively for nine years in the Norwegian Prescription Database (NorPD), capturing dispensed prescription drugs for sleep problems and anxiety/depression during the study period. Prescriptions are issued by medical doctors after a clinical assessment of the patients’ symptoms and are used as proxies for receiving treatment for sleep problems and/or anxiety/depression. The specific research objectives were to examine whether different constellations of parental risk, characterized by drinking quantity and frequency, mental health, and education, were associated with offspring’s subsequent treatment with prescription drugs during the 9-year study period for: (a) only sleep problems, (b) both sleep problems and anxiety/depression, and (c) only anxiety/depression.

## Methods

### Design, data sources and sample

We combined self-reports obtained from offspring and their parents who participated in the adolescent and adult version of the Nord-Trøndelag Health Studies [44, 45] in Norway in 2006–2008 (HUNT and Young-HUNT 3), with offspring registry data obtained from NorPD from 2008 to 2016. HUNT and Young-HUNT surveys are general population health surveys carried out in Nord-Trøndelag County in Norway, in which adults 20 and older, and all adolescents between 13 and 19 were invited to participate. The response rates for the surveys ranged from 54.1–82.7% [44–46]. For further information about response rates, handling of nonparticipation, and reasons for nonparticipation, please see [44–47]. Our sample included 8,774 adolescents (50.2% male) from 6,696 2-parent families, with mean age 16.05 years at young-HUNT participation. The HUNT surveys were conducted in an area that is mostly rural, with a lack of large cities, but the area is still considered to be fairly representative of Norway as a whole regarding geography, economy, industry, sources of income, age distribution, morbidity and mortality [45].

The HUNT and Young-HUNT health surveys constituted the study baseline and included information about exposures and covariates, while the 9-year longitudinal follow-up on outcomes of interest -- i.e., dispensation of any prescription drugs for sleep problems, and anxiety/depression in offspring --was done through NorPD.

Statistics Norway provided information on educational attainment and family identifier numbers for linkage on the family level.

We only included families where adolescent offspring and both parents had complete HUNT reports. We used a two-parent sample in order to isolate / identify the associations between exposure and outcomes in a sample not associated with multiple additional risk factors, including single-parent families.

All study participants provided written informed consent (or assent if younger than 16 years old). The study was approved by the Norwegian Data Protection Authority (# 38949) and the Regional Committees for Medical and Health Research Ethics (#2014/867).

### Measures

#### Exposures

The primary exposures were based on previously identified constellations of maternal and paternal risk factors based on parental education, alcohol consumption frequency and amount, and mental health [17]. The HUNT survey items included drinking frequencies “How many times a month do you normally drink alcohol?”. Drinking quantity was phrased as “How many servings of beer, wine or spirits do you usually drink in the course of two weeks?”. Parents reported the actual number of consumed drinks (cans/bottles of beer, glasses of wine and shots of liquor). Frequency responses were recoded to reflect mid-points of the original categories and quantity responses were summed to obtain total alcohol intake. To aid interpretation, estimates were rescaled to average weekly drinking quantities and frequencies. Parental mental health was measured using the Hospital Anxiety and Depression Scale (HADS). The sum scores translate to 0–7, normal; 8–10, mild symptoms; 11–14, moderate symptoms; and 15–21, severe symptoms. The HADS scale is considered a reasonably valid screening instrument in Norwegian samples [48]. The number of years of completed education for both parents were obtained from Statistics Norway. In a previous report we used these three parental risk factors to identify mutually exclusive parental risk constellations with latent profile (LP) analysis approach [17]. The latent profile (LP) analysis was conducted using Mplus with a default MLR estimator for all available data to classify family risk profiles based on indicators of parental drinking, mental health, and years of education at the time of each child’s participation in the Young-HUNT [17]. Table 1 provides a conceptual overview of the five identified parental risk constellations. LP1 was characterized by low education for both parents; LP2 by multiple risks in both parents: low education, mental-health symptoms indicating mild disorder and weekly binge drinking -approximately 4 alcohol units once per week for women, and approximately 11 alcohol units twice per week for men; LP3 by low overall risk in both parents: some higher education, good mental health, and infrequent low-quantity drinking. The only potential risk factor in LP4 was weekly binge drinking in both parents - about 4 alcohol units per drinking occasion twice per week for women, and about 5 alcohol units twice per week for men. LP5 have multiple risk factors: frequent and high-quantity drinking in both parents and mental-health symptoms suggesting mild disorder in fathers.

**Table 1:** Conceptual description of the parental risk constellations (Latent Profiles)

	Latent profile 1	Latent profile 2	Latent profile 3 (reference)	Latent profile 4	Latent profile 5
Characteristic	Low Education	Multiple risks: low education, binge drinking and mental health symptoms in both parents	Low overall risk	Binge drinking in both parents	Multiple risks: frequent heavy drinking both parents, mental health symptoms in fathers
**Participants**, ***n*** (%)					
Family ^a^	4,857 (69.1%)	194 (2.8%)	1,444 (20.5%)	473 (6.7%)	61 (0.9%)
Children	5,966 (68.0%)	246 (2.8%)	1,884 (21.5%)	598 (6.8%)	79 (0.9%)
**Education (years)** ^b^					
Maternal	**< 12**	**< 12**	> 12	> 12	> 12
Paternal	**< 11**	**< 12**	> 14	> 12	> 12
**Maternal drinking (weekly)** ^**c, d**^					
Average quantity	1 drink	**3.92 drinks**	1.25 drinks	**4.1 drinks**	**6.5 drinks**
Average frequency	0.4 days	0.95 days	0.5 days	**2.3 days**	**5.4 days**
**Paternal drinking (weekly)** ^**c, d**^					
Average quantity	2 drinks	**11.2 drinks**	2.3 drinks	**4.8 drinks**	**6.6 drinks**
Average frequency	0.7 days	1.9 days	0.9 days	**2 days**	**3 days**
**Mental health (HADS Score)** ^**c, e**^					
Maternal	Normal range	**Mild symptoms**	Normal range	Normal range	Normal range
Paternal	Normal range	**Mild symptoms**	Normal range	Normal range	**Mild symptoms**

*Notes*: The original Latent profile analysis procedures utilized all indicators in their original format [17]; to aid interpretation, we re-scaled the estimates to show average weekly drinking quantities and frequencies. Elevated levels of parental risk factors are shown in **bold**. ^a^Some families had multiple children, therefore the number of children is greater than the number of families for each LP. ^b^From the Statistics Norway records. ^c^From parental self-reports. ^d^ Quantity = number of glasses of beer, wine, or liquor reported in HUNT surveys. ^e^ HADS (14-item Hospital Anxiety and Depression Scale) is commonly used to screen for anxiety and depression. Sum scores translate to the following diagnostic categories: 0–7 normal, 8–10 mild, 11–14 moderate, and 15–21 severe symptom

#### Outcomes

We examined dispensation of prescription medication for sleep problems and anxiety/depression as annually recorded in NorPD between 2008 and 2016. NorPD covers information on prescription drugs dispensed at pharmacies in Norway (for details please see [49]). Offspring participants were categorized into four mutually exclusive groups: (1) neither sleep nor anxiety/depression medications dispensed during the study period; (2) sleep medications (but not any anxiety/depression medication) dispensed at least once during the study period; (3) anxiety/depression medications (but not any sleep medication) dispensed at least once during the study period; and (4) both sleep and anxiety/depression medications dispensed at least once during the study period. Table 2 shows an overview of ATC codes used to identify prescription drugs for sleep problems and/or anxiety/depression.

**Table 2:** Overview of registry entries on prescription drugs dispensed to adolescents/young adults for sleep problems and anxiety/depression

Prescription drugs for sleep problems		Prescription drugs for anxiety/depression	
*ATC* ^*a*^ *codes* ^*b*^		*ATC* ^*a*^ *codes*	
N05CD02	Nitrazepam	ATC codes starting with N05B	Anxiolytics
N05CD03	Flunitrazepam	ATC codes starting with N06A	Antidepressives
N05CF01	Zopiclone		
N05CF02	Zolpidem		
N05CH01	Melatonin^c^		

^a^Anatomical Therapeutic Chemical. ^b^N05CM Passionflower were originally included, but had not been dispensed to any individuals in our sample. ^c^Melatonin was only available through prescription in Norway during the study period (2008–2016). It was made available for purchase without prescription in 2020.

#### Covariates

Offspring demographic characteristics included: age at baseline survey participation, age at first registry follow-up in 2008, and sex.

Frequencies of offspring’s current sleep problems during adolescence were assessed with the two Young-HUNT questions: “Had difficulty falling asleep in the evening” and “Woke too early and couldn’t fall asleep again”. Because of the low frequencies in the two most severe categories, the original response options of “never”, “sometimes”, “often” and “almost every night” were recoded such that “often” and “almost every night” categories were collapsed. A composite variable capturing the entirety of early sleep problems was computed as a sum of the two original items.

Severity of offspring’s current mental-health symptoms during adolescence were assessed with the five-item Hopkins Symptoms Checklist (SCL-5) administered as part of the Young-HUNT survey [50]. SCL-5 scores were categorized to reflect the top 25% of the distribution versus the rest. Missing responses (n = 138) were retained as a separate category to prevent loss of data. Sensitivity and specificity for SCL-5 have been estimated at 82% and 96% [50].

Adolescent alcohol use has also been associated with sleep problems [51]. We therefore included early indicators of alcohol consumption as a covariate. Number of alcohol units (cans/bottles of beer, glasses of wine, and shots of liquor) consumed during a typical two-week period were categorized to reflect typical bi-weekly alcohol consumption of no alcohol use, 1–5 units, and > 5 units. Missing responses (n = 770) were retained as a separate category to prevent loss of data.

As in our previous studies [17, 52, 53], we did not use any advanced methods for handling missing data for these covariates; missing data on the two categorical variables, self-reported mental health and early indicators of alcohol consumption, were coded as separate categories of “no valid report” to prevent data loss. Therefore, our analytical *n* was not reduced, and all reported estimates are based on *N* = 8,773 offspring.

### Statistical analyses

We reported basic descriptive statistics for the sample, and for exposure and outcome variables. We used multinomial logistic regression to model the odds of the outcomes -- that is, offspring’s use of no prescription medication, sleep medications only, anxiety/depression medications only, and both sleep and anxiety/depression medication during the follow-up period -- as a function of early parental risk constellations, with “no prescription” as a base comparison group. All analyses were conducted using the STATA *mlogit* command. Models were estimated with clustered robust errors to account for within-family nesting - cases in which multiple children in the same family. All models adjusted for all covariates; reported were thus adjusted Relative Risk Ratios (aRRR), commonly interpreted as Odds Ratios (OR), with corresponding 95% CI.

## Results

Table 3 provides and overview number and percentage of adolescent and adult offspring in each outcome group.

**Table 3:** N and percent (%) of adolescent and adult offspring in each outcome group

Outcome groups, prescription drugs for:	N	%
Neither sleep problems nor anxiety/depression	7007	79.87
Sleep problems, but not anxiety/depression	381	4.34
Both sleep problems and anxiety/depression	460	5.24
Anxiety/depression, but not sleep problems	925	10.54

Results from the multivariate multinomial regression model are reported in Table 4. Compared to the lowest risk constellation, no other parental constellation was significantly associated with the risk of offspring receiving prescription medication for only sleep problems during the study period. Compared to the lowest overall parental risk constellation (LP3), two LPs were significantly associated with greater risk of offspring being dispensed both sleep and anxiety/depression prescription drugs during the study follow-up. Specifically, offspring in LP2, with binge drinking, mental health symptoms and low education in both parents (OR 1.90, CI 1.06;3.42) and offspring in LP5, with frequent drinking in both parents and mental health symptoms in fathers (OR 3.32, CI 1.49;7.39). Finally, compared to the lowest overall parental risk constellation, the risk constellation characterized by low education in both parents (LP1) was significantly associated with greater risk of offspring receiving prescription medication for only anxiety/depression during the study period; OR = 1.25, CI 1.05;1.49.

**Table 4:** Dispensed prescription drugs (2008-16): only sleep problems, only anxiety/depression, and both sleep problems and anxiety/depression as a function of parental risk constellations and control variables

		Sleep prescription drugs			Anxiety/depression prescription drugs			Both sleep and anxiety/depression prescription drugs		
	n (%)	OR	95% CI		OR	95% CI		OR	95% CI	
*Parental risk constellation/latent profiles* **(unadjusted)**										
LP1: low education	5966 (68.0)	0.99	0.77	1.27	**1.27**	1.06	1.51	**1.33**	1.03	1.71
LP2: multiple risks – binge drinking, mental-health symptoms, and low education in both parents	246 (2.8)	0.84	0.41	1.70	1.20	0.76	1.88	**1.80**	1.01	3.20
LP3: reference, low overall risk	1884 (21.5)									
LP4: binge drinking in both parents	598 (6.8)	0.90	0.57	1.42	0.78	0.55	1.11	1.20	0.79	1.83
LP5: multiple risks – frequent heavy drinking both parents, mental-health symptoms fathers	79 (0.9)	0.59	0.14	2.47	1.31	0.65	2.62	**2.21**	1.00	4.91
Sex (male)	4406 (50.2)	**0.78**	0.63	0.97	**0.58**	0.50	0.67	**0.65**	0.53	0.79
	**Mean (SD)**									
Age at Young-HUNT baseline	16.05 (1.79)	0.95	0.89	1.02	0.98	0.93	1.03	**0.89**	0.83	0.96
Age at first registry follow-up in 2008	23.80 (5.68)	**1.06**	1.04	1.08	**1.04**	1.02	1.05	**1.08**	1.06	1.11
Adolescent early sleep problems (sum 0–3)	0.95 (0.93)	**1.24**	1.12	1.38	**1.10**	1.03	1.19	**1.35**	1.23	1.48
	**n (%)**									
*Adolescent offspring mental health (SCL-5)*										
Normative range (reference)	6660 (75.9)									
Clinical-level range	1975 (22.5)	**1.70**	1.34	2.17	**1.99**	1.70	2.34	**1.55**	1.25	1.93
No response	138 (1.6)	**2.86**	1.44	5.65	**2.60**	1.62	4.17	1.04	0.41	2.61
*Adolescent alcohol consumption usual 2-week period*										
None (reference)	4294 (48.9)									
1–5 drinks	1902 (21.7)	1.04	0.77	1.39	1.01	0.83	1.23	1.16	0.89	1.52
more than 5 drinks	1807 (20.6)	1.14	0.83	1.57	1.05	0.84	1.30	**1.45**	1.08	1.95
no valid report	770 (8.8)	0.96	0.65	1.42	1.11	0.86	1.42	**1.48**	1.07	2.05
*Parental risk constellation/latent profiles* **(adjusted)**										
LP1: low education	5966 (68.0)	0.97	0.75	1.25	**1.25**	1.05	1.49	1.29	0.99	1.66
LP2: multiple risks – binge drinking, mental-health symptoms, and low education in both parents	246 (2.8)	0.87	0.43	1.78	1.25	0.78	1.99	**1.90**	1.06	3.42
LP3: reference, low overall risk	1884 (21.5)									
LP4: binge drinking in both parents	598 (6.8)	1.04	0.66	1.65	0.85	0.60	1.21	1.49	0.96	2.31
LP5: multiple risks – frequent heavy drinking both parents, mental-health symptoms fathers	79 (0.9)	0.79	0.19	3.29	1.65	0.84	3.25	**3.32**	1.49	7.39

## Discussion

In this large prospective cohort study, we found that parental risk constellations were differently associated with receiving medications for sleep problems in individuals depending on whether they also received medications for anxiety/depression during the study period. Offspring in the four risky constellations were not more likely than offspring in the overall low-risk group to receive sleep medications when they did not also receive anxiety/depression medication. Two risk constellations were associated with increased risk of receiving sleep medication when the offspring also received anxiety/depression prescription drugs: the first is families with frequent drinking in both parents and mental health symptoms in fathers (LP5), and the second is binge drinking, mental health symptoms and low education in both parents (LP2). Offspring from families with low education in both parents were more likely to receive only prescription drugs for anxiety/depression and not for sleep problems (LP1).

This was the first study to use a cluster approach to study the association between parental drinking, mental health and education, and subsequent offspring prescription drug use for sleep problems, which enabled us to disentangle the role of parental risk constellations in predicting prescription drug use for sleep problems with and without preceding, co-occurring or following anxiety/depression prescription drug use. We found that parental risk constellations were not associated with the likelihood of being dispensed sleep medication when this prescription was not preceded or followed by medication for anxiety/depression. This is in contrast to findings in previous studies that showed an effect of parental drinking, parental socioeconomic status, parental mental health on other offspring sleep outcomes [18–20, 26]. For instance, two US studies showed an association between parental drinking on preadolescents sleep problems, measured by actigraphs, and that the effect was stronger in families with low socioeconomic status [18, 19]. One of these studies controlled for early indicators of anxiety and depression at baseline [18], the other did not [19], and neither of the studies addressed whether sleep problems at follow-up occurred in conjunction with anxiety/depression. Our findings contrast those of a recent study from the US, which examined the association between parental depression and sleep problems in preadolescent offspring [26]. Offspring whose parents had depression were at increased risk of sleep problems compared to offspring of non-depressed parents [26]. Further, parent-reported lower socioeconomic status was also associated with increased risk of offspring sleep problems [26]. The study controlled for offspring anxiety and depression symptoms, and to avoid confounds, sleep items were removed from the anxiety and depression measures [26]. Our findings also contrast those of a Canadian study which showed that adolescents low subjective socioeconomic position, were associated with lower self-reported sleep quality, also after controlling for adolescent anxiety and depression [20].

Our findings that two parental risk constellations were associated with increased risk of being dispensed sleep medication, when also being dispensed anxiety/depression prescription drugs, could be interpreted as supporting the abovementioned studies, which showed an association between parental drinking, mental health, and SES, on offspring sleep problems [18–20, 26]. However, most adolescents with depression, also have sleep problems, and those who do, tend to have more severe depression [33, 54]. A plausible explanation, therefore, is that since none of the parental risk constellations predicted receiving sleep medications only, what the parental risk constellations predict are the more severe cases of anxiety/depression, which are also dispensed prescription drugs for sleep problems. The finding that only one parental risk constellation was associated with receiving only anxiety/depression drugs, adds support to the interpretation that parental risk factors are primarily associated with the most severe cases of anxiety/depression, which typically occur in conjunction with sleep problems. This sheds light on our previous work, where we found an association between some parental risk constellations and subsequent treatment for anxiety/depression in offspring [17]. Seen in the light of the finding from the current report, the associations observed in our previous work were likely largely driven by more severe cases, with both sleep problems and anxiety/depression.

Our study extends and adds nuance to the research on parental risk factors as predictors of offspring sleep problems. Taken together, our findings suggest that parental risk constellations of drinking patterns, mental health, and education, are not associated with offspring receiving prescription drug treatment for sleep problems, in cases where they do not also receive medications for anxiety and/or depression. The findings underscore the importance that studies primarily examining sleep problems, control for anxiety and depression to tease out the associations pertaining to sleep. As our results show, there may otherwise be a risk that associations that stem from anxiety/depression be misattributed to sleep. This problem does not extend to anxiety/depression research, where sleep symptoms are included among the symptoms of anxiety/depression. Future research should develop interventions that educate parents about the effect their alcohol use and mental health may have on their offspring (increased risk of the combination of sleep problems and anxiety/depression), and provide support and advice on self-care and reducing alcohol use; this can alleviate the problems experienced by the parents, which in turn will likely benefit their children. Prescription drugs remain the most frequently used treatment for sleep problems in Norway. GPs, adolescents, and young adults with sleep problems, and parents of adolescents with sleep problems should consider other alternatives to prescription drug treatment for sleep problems. For instance, GPs should advise on good sleep hygiene, including information about where patients can find additional resources. GPs should also provide information about alternatives, e.g., the possibility of attending courses and individual guidance sessions at community wellness centres. Further, recent studies have tested the effect of online CBT interventions for reducing sleep problems in a Norwegian setting; results showed that this is an effective approach in reducing the severity of symptoms associated with sleep problems [55, 56], suggesting that such online tools should be made widely available, and tried before prescribing prescription drugs for sleep problems.

### Methodological considerations

Major strengths of the study include the large sample size, the prospective design, practically no attrition, the use of person-centered approaches that identified parental risk profiles based on information obtained from both mothers and fathers. The study has several limitations, that should be taken into consideration when interpreting the results. Our sample consisted of two-parent families where mothers, fathers and adolescents participated in HUNT. While this limits generalizability to other family constellations, an advantage with the approach is that it steers clear of the single data source limitations and biases that previous studies are fraught with, e.g. information about parental characteristics is provided by the adolescents only, or by the adolescent and just one parent [57]. We used self-report data at baseline, this may have resulted in underreporting, inaccurate recall and selective reporting [58]. In addition, even though registry linkages ensured no loss in terms of follow-up data, not all participants reported their alcohol use and/or depression/anxiety symptoms during adolescence when they completed the HUNT survey. We aimed to preserve these cases by simply treating them as a separate “no valid report” category in all analyses, but this may have somewhat biased the estimates [59]. However, it should be noted that the addition of covariates did not substantially alter the associations between primary exposures and outcomes in our model, as evident in comparable estimates obtained from the unadjusted and adjusted model. Our registry-based outcomes are conservative as they only include individuals that have self-selected into seeking help for, been diagnosed with a disorder, and received prescription drugs for treating these disorders. We used NorPD to capture the outcomes of interest instead of healthcare utilization databases. The rationale is that in consultations with general practitioners (GPs), patients often raise several health issues, and while some GPs records all the issues in the primary healthcare database, many record only the primary reason for the consultations, thus if sleep problems, or anxiety/depression were not the primary reason, these problems are not captured in the database. Thus, for looking at anything but primary diagnoses/main reason for contact, the primary healthcare database is less reliable than the NorPD, which captures all prescriptions dispensed. For our purpose, NorPD is therefore more reliable, because all who receive prescription drugs are issued prescriptions after consultation and diagnosis by a medical doctor. Consequently, this approach does not capture the population prevalence of sleep problems and/or anxiety/depression, which is likely substantially higher, only cases that are both diagnosed and receive treatment with prescription drugs. Further, while prescription drugs are, by far, the most frequently used treatment for sleep problems in Norway, several support resources for sleep problems are not picked up in a study such as ours, e.g., online interventions to support sleep [55], and individual consultations, and courses on how to achieve better sleep at wellness centers in the municipalities. It may be that these resources are used by a different group of individuals that struggle with sleep problems than those that receive treatment with prescription drugs. Unfortunately, given the nature of our data, we cannot say whether this is the case.

Sleep problems, as captured in NorPD, may include cases that receive prescription medication for sleep problems due to transient situational factors, such as stress, bereavement, relationship problems. It seems plausible that parental factors are less important for such problems. However, given the nature of the of the data, we have no way of identifying whether the prescription drug use was due to transient situational factors or not. Further, while we know that patients were dispensed the prescription medications, we do not know whether they used them. It should also be noted that some of the prescription drugs used to treat anxiety/depression are also sleep inducing; thus, some patients that experienced both depression/anxiety and sleep problems, who received these prescription drugs, may have had both of their problems solved with one medication. The study does not account for off-label use of other types of medications that may have been used to alleviate sleep problems, or use of non-prescription drugs. Finally, we use the long follow-up time as a rationale for separating between sleep problems with and without preceding, co-occurring or following anxiety/depression. However, nine years is not lifetime, and we can only generalize to the years for which we have data. It is possible that study participants received prescription drugs that contradicts this assumption after follow-up ended in 2016, and for the oldest cohort, during the years preceding registry follow-up start.

## Conclusion

The role of different constellations of parental risk, characterized by drinking quantity and frequency, mental health, and education played in predicting offspring’s subsequent sleep problems is nuanced. While no parental risk constellations were associated with increased risk of offspring receiving prescription drugs for only sleep problems, during the nine-year study period, two were associated with increased risk that offspring received prescription drugs for both sleep problems and anxiety/depression. The findings underscore the importance that sleep studies control for anxiety/depression.

## Data Availability

Norwegian legislation prohibits sharing data from this project. However, other researchers that wishes to use HUNT and Registry data used in this project can apply to the Regional Committees for Medical and Health Research Ethics (REK). Applicants and projects must fulfill requirements in Norwegian regulations and laws concerning research and protection of personal information (GDPR), and project proposals must also be approved by the data owners. Guidelines for application to REK can be found here: https://rekportalen.no/#hjem/home. REK approval must be in place before researchers apply to data owners listed below. Guidelines for applications to HUNT data are found at: https://www.ntnu.edu/hunt/data. Guidelines for application to Statistics Norway data are found at: https://www.ssb.no/en/data-til-forskning/utlan-av-data-til-forskere/soknad. Guidelines for application for The Norwegian Prescription database data are found at: https://helsedata.no/en/.

## List of Abbreviations

aRRR: Relative Risk Ratios
SCL-5: Hopkins Symptoms Checklist
CI: Confidence Interval
HADS: Hospital Anxiety and Depression Scale
HUNT: The Nord-Trøndelag Health Studies
LP: Latent Profile
NorPD: the Norwegian Prescription Database
OR: Odds Ratio
SES: Socioeconomic status
